# Sex differences at the platelet–vascular interface

**DOI:** 10.1007/s11739-022-02994-y

**Published:** 2022-05-16

**Authors:** Annamaria Sabetta, Ludovica Lombardi, Lucia Stefanini

**Affiliations:** Department of Translational and Precision Medicine, Sapienza University of Rome, Viale dell’Università, 37, 00185 Rome, Italy

**Keywords:** Platelets, Sex, Thromboinflammation, Vascular disease

## Abstract

Platelets are multifunctional cells that ensure the integrity of the vascular wall and modulate the immune response at the blood/vascular interface. Their pathological activation results in both thrombosis and inflammation and implicates them in the pathogenesis of vascular disease. Vascular diseases are sexually dimorphic in terms of incidence, clinical presentation, outcome, and efficacy of anti-platelet therapy. We here provide an overview of what is known about the role of platelets in the initiation and progression of vascular diseases and summarize what is known about the sex differences in platelet reactivity and in the thromboinflammatory mechanisms that drive these diseases, with a particular focus on atherosclerosis, obstructive and non-obstructive coronary artery disease, and ischemic stroke. Understanding the sex differences at the platelet–vascular interface is clinically relevant as it will enable: (1) to design new therapeutic strategies that prevent the detrimental effects of the immune-modulatory function of platelets taking sex into account, and (2) to evaluate if sex-specific anti-platelet drug regimens should be used to reduce the risk not only of thrombosis but also of vascular disease progression.

## Platelets as regulators of vascular barrier function

Platelets are the first cells to sense and respond to perturbations of the vascular barrier function. Their role, however, is not limited to sealing injuries. Platelets also (1) ensure the integrity of the vascular wall during homeostasis and at sites of inflammation, (2) provide first line defense against pathogens and modulate the immune response, and (3) promote tissue repair [1].

### Classical hemostasis

Platelets are geared with a signaling machinery that enables them to release bio-active compounds and to become adhesive very rapidly. Traditionally these highly specialized features have been studied in relation to their ability to prevent blood loss at a site of injury (*hemostasis*). Lesions that break the integrity of the endothelial lining expose components of the sub-endothelial matrix and of damaged cells, such as collagen, Willebrand factor (VWF) and tissue factor. Within seconds, the interaction between glycoprotein (GP) Ib-IX-V and collagen-bound VWF decelerates platelets at the injury site and tissue factor stimulates the coagulation cascade. Collagen and locally generated thrombin stimulate platelets to activate integrins and secrete intracellular granules. Active integrins support stable adhesion to the exposed matrix and aggregation with other active platelets. Molecules secreted from the granules, e.g., adenosine diphosphate (ADP) and serotonin, and newly synthesized mediators, (e.g., thromboxane A_2_), provide a positive feedback and recruit more platelets from the blood stream. Furthermore, serotonin and thromboxane A_2_ limit blood loss by inducing vasoconstriction. Thrombin cleaves fibrinogen into fibrin that polymerizes into a three-dimensional web and binds aggregated platelets to form a stable hemostatic plug.

**Fig. 1:**
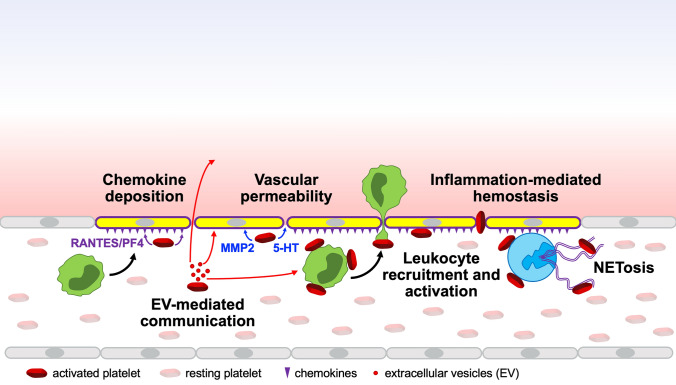
Platelets are multifunctional cells that ensure the integrity of the vascular wall and modulate the immune response at the blood/vascular interface. Platelets are always the first cells to sense any vascular anomaly and bind to activated endothelial cells (yellow). Once recruited, platelets regulate vascular inflammation by controlling deposition of chemokines, vascular permeability, leukocyte recruitment and activation, and inflammation-mediated hemostasis. They can also modify the phenotype and function of vascular smooth muscle cells, endothelial cells and leukocytes through extracellular vesicle (EV)-mediated communication

A similar sequence of events is believed to happen on a ruptured atherosclerotic plaque, with the difference that a pathological thrombus grows within the vessel lumen and limits the flow of blood to tissues and organs causing ischemia (*thrombosis*). However, studies in the past 20 years demonstrate that platelets have pathophysiological functions that reach far beyond hemostasis and thrombosis (Fig. 1) [2].

### Inflammation-mediated hemostasis

Numerous studies demonstrate that platelets ensure vascular integrity also when the vessel is intact, but the endothelial barrier is dysfunctional because of inflammation (reviewed in Ref. [3])*.* Clinically this is evident when thrombocytopenic patients bleed during viral infections, or cancer, upon ischemia–reperfusion injury, or UV exposure. The bleeding occurs at sites of leukocyte extravasation and can be prevented by anti-inflammatory drugs irrespective of improvement in platelet counts [4]. While the role of platelets in classical hemostasis requires rapid activation of integrin-mediated aggregation, in the setting of inflammation, integrin-mediated aggregation is dispensable and platelets are activated via signaling mediated by glycoprotein VI (GPVI), C-type lectin-like receptor 2 (CLEC-2) or GPIbα, depending on the tissue [3].

Interestingly, a recent study has shown that platelets are needed to prevent vascular leakage also in homeostatic conditions, in the absence of injury or inflammation. In this setting, dense granule secretion and GPVI, but not CLEC-2, signaling are required to maintain vascular barrier function [5].

### Immuno-thrombosis

The breakdown of vascular barrier function enables the invasion of microorganisms and might provoke tissue infections. Platelets actively participate in host defense in a process called *immuno-thrombosis*. They aggregate to limit pathogen spread in the blood, directly capture and kill pathogens, stimulate innate immune responses, such as the formation of neutrophil extracellular traps (NETs), and prime the adaptive immune response (reviewed in Ref. [6]). Platelet granules contain anti-microbial factors and positive and negative regulators of inflammation, that control vascular permeability, attract leukocytes, and modulate immunity. α-granule release also results in the surface exposure of P-selectin, a platelet adhesion receptor that binds PSGL-1 (P-Selectin Glycoprotein Ligand 1) on leukocytes and endothelial cells and facilitates immune cell recruitment at sites of endothelial activation. Conformationally active αIIbβ3 integrins mediate, not only homotypic platelet-platelet interactions, but also heterotypic interactions with leukocytes, endothelial cells, and pathogens. GPIbα participates in leukocyte recruitment by binding integrin αMβ2 (Mac-1) and contributes to bacterial clearance by binding complement-opsonized bacteria. Moreover, platelets are equipped with pattern recognition receptors (TLRs 1–9, NLRP3, NOD2, DC-SIGN, CLEC-2) that sense pathogen-associated molecular patterns (PAMPs) and damage-associated molecular patterns (DAMPs) and with immuno-receptors (FcγRIIA, FcεRI, and FcαRI) that activate platelets in response to immune complexes.

The dysregulated activation of platelets in inflammatory conditions, such as uncontrolled infections, autoimmune disease and clonal haematopoiesis of indeterminate potential, may cause pathological thrombosis and tissue ischemia, in a process called *thrombo-inflammation.*

## The role of platelets in the pathogenesis of vascular disease

Most vascular diseases can be considered thrombo-inflammatory conditions, in which thrombosis is just the final stage. Because of their ability to regulate the immune response and vascular integrity (Fig. 1), platelets are not just the building blocks of thrombi, but also contribute to the initiation and amplification of vascular disease.

### Atherosclerosis

The central role of platelets in stimulating vascular disease is illustrated by atherosclerosis. Atherosclerosis is a chronic inflammatory vascular disease characterized by the accumulation of monocytes/macrophages and lymphocytes in the innermost layer of large arteries (tunica intima). Atherosclerotic lesions preferentially build in areas where blood flow is disturbed. Shear stress in combination with unhealthy life-style habits (smoking, high-fat diet, poor physical activity) promotes endothelial activation. Platelets are always the first cells to sense any vascular anomaly and bind to the activated endothelium before the development of manifest atherosclerotic lesions [7]. Once recruited, platelet contributes to atherogenesis in 4 ways (reviewed by Coenen et al. [8]).

First, platelets rolling on activated endothelial cells deposit chemokines CCL5 (RANTES) and CXCL4 (platelet factor 4, PF4) that accelerate leukocyte recruitment on the inflamed vascular wall [9].

Second, platelets upregulate the adhesive properties of endothelial cells and increase vascular permeability to facilitate leukocyte transmigration. A recent study demonstrated that platelet MMP2 (Matrix Metallo Peptidase 2) cleaves endothelial PAR1 and promotes expression of VCAM (Vascular Cell Adhesion Molecule) on endothelial cells, which results in transmigration of pro-inflammatory monocytes and plaque growth [10]. Moreover, platelets are the main source (outside of the brain) of serotonin that is elevated in coronary artery disease (CAD) patients [11] and increases vascular permeability [12].

Third, platelets regulate leukocyte migration and function. One of the transcripts most highly expressed in myocardial infarction (MI) patients compared to stable CAD is MRP-14 [13], that is released by platelets as a dimer with MRP-8 (MRP8/14, calprotectin), to stimulate monocyte and neutrophil migration and activation [14]. Increasing plasma concentrations of MRP-8/14 among healthy individuals predict the risk of future cardiovascular events [13]. Serotonin can also drive neutrophil recruitment to inflamed tissues [15] and modulate T lymphocyte function [16]. Moreover, platelets have been shown to promote monocyte trafficking toward the plaque and skewing of plaque macrophages toward an inflammatory phenotype [17], by decreasing the ratio of SOCS1:SOCS3 (Suppressor Of Cytokine Signaling 1 and 3), transcriptional regulators cytokine signaling [18].

Last, platelets release extracellular vesicles (EVs) that mediate intercellular communication. Because of their small size, EVs can also affect macrophages and smooth muscle cells in the extra-luminal spaces.

### Deep vein thrombosis

Platelets also contribute to the initiation of deep vein thrombosis (DVT) (reviewed by Budnik [19]). In this context, the endothelium is activated by blood flow stagnation and hypoxia, and the initial recruitment of single platelets is supported by GPIbα binding to VWF released from Weibel Palade bodies (endothelial cell granules). Moreover, hypoxia promotes the loosening of endothelial cell–cell junctions. Thus, platelets are allowed to penetrate the sub-endothelial space and activate through CLEC-2 that binds podoplanin overexpressed in tunica media and adventitia (middle and external layers of the venous wall, respectively) [20]. Activated platelets stimulate coagulation through the secretion of polyphosphates (PolyP) and recruit/activate leukocytes by releasing various DAMPs, like HMGB1 and MRP8/14. Recruited monocytes foster coagulation by exposing tissue factor. Recruited NETs [21], web-like structures contain de-condensed DNA, histones and the content of neutrophil secretory granules. In turn, NETs stimulate coagulation, platelets and endothelial cells and form a scaffold that supports the growth of the venous thrombus.

### Ischemic stroke

In the setting of stroke, platelets are not just components of the clots obstructing the vessels, but also contribute to stroke progression (reviewed by Stoll et al. [22]). They bind to leukocytes shortly after the event and modulate their function. Platelets promote the release of neutrophil extracellular traps that, in turn, increase the severity of the brain injury by damaging the blood–brain barrier [23] and by impairing revascularization and vascular remodeling after stroke [24].

After the clot is removed (recanalization by pharmacological thrombolysis and/or mechanical thrombectomy), platelets contribute to stroke progression by orchestrating ischemia–reperfusion injury together with T cells [22]. Platelets are engaged to the site of endothelial damage via GPIbα-VWF interaction, become activated in a GPVI-dependent manner and release pro-inflammatory and pro-coagulant EVs and PolyP. Moreover, CD84 is shed from the surface of activated platelets and soluble CD84 stimulates CD4^+^T cell motility [25]. In the setting of transient ischemia, CD4^+^ T regulatory cells (Treg) are responsible of aggravating microvascular dysfunction and ischemia. Conversely, in permanent ischemia, Tregs are neuroprotective and other T cell subtypes exert detrimental effects by migrating into the brain parenchyma [22].

Integrin-mediated platelet aggregation is dispensable for ischemia–reperfusion injury and stroke progression, but it is required for hemostasis in the brain. Therefore, use of anti-platelet drugs after recanalization is dangerous as it increases intracranial bleeding without protecting from stroke progression [26].

### Microvascular dysfunction

While the role of platelets in promoting vascular inflammation in large vessels is well established, less is known about the role of platelets in amplifying microvascular inflammation and dysfunction. A significant correlation exists between platelet reactivity and coronary microvascular function at the time of percutaneous coronary intervention [27]. In a mouse model of ischemia/reperfusion injury, inhibition of platelets with cilostazol, a PDE3 inhibitor that increases intracellular cyclic AMP level like endothelial-derived prostaglandin, reduced microcirculatory and organ damage, possibly by inhibiting the interaction of platelets with the injured endothelium [28]. Moreover, platelets have been implicated in leukocyte recruitment to venules inflamed by colitis [29] and in the impairment of vasodilation in venules inflamed by hypercholesterolemia [30].

## Sex differences in platelet reactivity

**Fig. 2:**
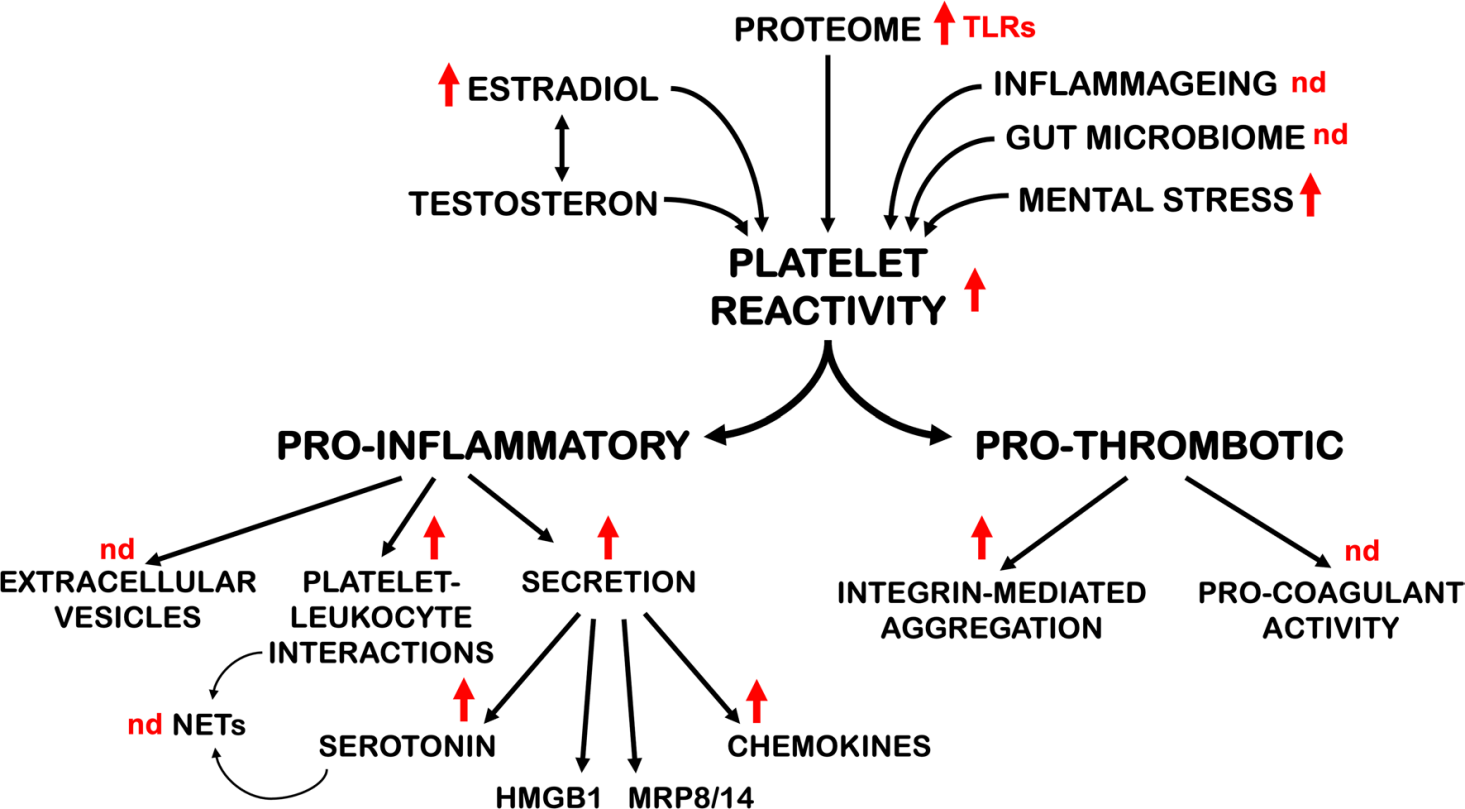
Mechanisms and consequences of sex differences in platelet reactivity. The increased platelet reactivity of women (indicated with red arrows, nd: not determined) is caused by intrinsic and extrinsic differences between males and females and determines the upregulation of both the pro-thrombotic and the pro-inflammatory functions of platelets

Sexual dimorphism in platelet reactivity was recognized almost 50 years ago [31]. It is now well established that, at all ages, platelets from women are more responsive to agonist stimulation than platelets from men. Platelet reactivity is enhanced in response to all agonist tested, in particular after stimulation of the thrombin receptor PAR1 [32, 33]. Women also have a higher platelet count [34]. However, their increased platelet responsiveness is observed even after adjusting the count to the same cell concentration as men [35]. The early studies evaluated platelet reactivity by standard aggregation in platelet-rich plasma and a correlation between the percentages of aggregation with plasma fibrinogen concentration was noted. Nevertheless, later studies with washed platelets [32] confirmed the increased female platelet reactivity, suggesting that it is an intrinsic property of female platelets. At the molecular level, flow cytometry studies reveal that female platelets have a higher propensity to activate integrin αIIbβ3, to degranulate and expose P-selectin, and to form platelet–leukocyte aggregates [36]. Thus, the sexual dimorphism affects both the pro-aggregating and the immune-modulatory function of platelets (Fig. 2).

The higher platelet reactivity of women has been observed among healthy individuals, asymptomatic individuals with family history of premature CAD [37], outpatients with suspected MI [38] and in patients undergoing stenting [36]. Interestingly, in a cohort of post-MI patients (studied before the loading dose of P2Y12 antagonist), the platelet reactivity of women decreased after MI, but the reactivity of male platelets increased [39]. It is not known if this change in platelet responsiveness post-MI is due to the different pathobiology of MI among sexes or simply to the fact that female platelets are more reactive and become exhausted more easily during MI.

Despite the higher platelet responsiveness, women experience the same decrease in platelet reactivity after aspirin therapy compared with men, retaining modestly more platelet reactivity [40]. However, according to a more recent study, women, but not men, experience an attenuation of the aspirin effect after 4 weeks of therapy [41]. Conversely, in response to clopidogrel, women are more likely than men to have high-on-treatment residual platelet reactivity (HRPR) [42]. HRPR correlates with a higher platelet count. Thus, it is unclear if the higher basal platelet reactivity of women contributes to HRPR. In patients taking dual antiplatelet therapy, sex does not influence the prevalence of HRPR with the major antiplatelet agents ASA, clopidogrel or ticagrelor [43]. It must be noted however that, while P2Y12 inhibition is very effective in reducing fibrinogen binding in both sexes, P-selectin exposure is less inhibited especially in women, suggesting that there may be a sex difference in the ability of these drugs to inhibit the platelet pro-inflammatory functions.

### Intrinsic sex differences of platelets

Mechanisms responsible for sex differences in platelet function are largely unknown. Proteins, mRNAs, and miRNAs [44, 45] are differentially expressed by sex in platelets, supporting the concept that sex differences in platelet reactivity are, at least in part, an intrinsic property of platelets. Platelets derived from women express a higher proportion of transcripts encoding for the TLRs (Toll-like receptors) compared to men [44], and the expression level of platelet TLRs correlates with an increased concentration of soluble P-selectin, that is shed from the surface of activated circulating platelets. Conversely, expression of a TLR4-hyporesponsive variant (Asp229Gly), is associated with an increased risk of myocardial infarction in men but not in women [46]. These data might suggest that women platelets have an intrinsic higher propensity to be activated by inflammatory stimuli compared to men, but this speculation awaits further confirmation.

### Effect of sex hormones on platelet function

The fact that platelet reactivity changes along the menstrual cycle and after menopause suggests that it is affected, at least in part, by sex hormones. Platelets express the estrogen receptor β (ERβ) and the androgen receptor (AR) [47]. However, the reports on the effects of estrogens and testosterone on platelets have been controversial. In general, 17β-estradiol (the most biologically active estrogen) is believed to exert a protective effect on vascular disease, which is lost after menopause. Consistently, at least two studies show that in vitro administration of estradiol reduces platelet activation. In contrast, another work demonstrates that estradiol evokes a non-genomic signaling pathway, involving Src, Pyk2 and phosphatidylinositol 3-kinase, that has pro-aggregating effects by synergizing with thrombin signaling [48]. Studies on the difference of platelet reactivity before and after menopause and on the in vivo administration of hormone replacement therapy post-menopause, are also very contradictory (reviewed by Dupuis et al. [49]). The effect of hormone replacement therapy seems to depend on the dosage and on the timing of the administration.

In vitro incubation of testosterone with human platelets potentiates platelet aggregation equally well for both men and women [31], by augmenting the production of thromboxane A_2_ [50] and by reducing the production of nitric oxide [51]. In mice, supra-physiological doses enhance platelet activation and thrombopoiesis, while castration results in lower platelet aggregation. In humans, low levels of circulating testosterone have been associated with increased risk of atherosclerotic manifestations, and there have been many case reports of thrombosis in athletes that abuse of anabolic androgenic steroids [52].

A possible reason for the contradictory reports on the effect of sex hormones, may be that estradiol and testosterone are co-dependent and evoke synergistic effects. For this reason, the testosterone/estradiol ratio is considered a better tool than the absolute concentration of hormones to predict the effects of sex steroids on platelet reactivity and vascular disease. Among post-menopausal women, a higher testosterone/estradiol ratio is associated with an elevated risk for incident CVD events [53]. In men with severe atherosclerosis, low testosterone/estradiol ratio is related to increased systemic and plaque inflammation and predicts future cardiovascular events [54].

### Extrinsic determinants of platelet sexual dimorphism

The sex-specific contribution of platelets to vascular disease may also depend on extrinsic factors such inflammageing, the gut microbiota composition, and mental stress.

Aging is strongly associated with an increased risk of CVD. Platelets from both older men and women have a greater sensitivity to aggregation induced by classical agonists, as if platelets were pre-activated (reviewed by Le Blanc et al. [55]). In line with this, the proportion of circulating reticulated platelets, a surrogate marker of platelet turnover, increases with age [56], possibly because of increased replacement of consumed platelets.

One of the most documented changes during aging is the increase of chronic low-grade and systemic inflammation, a condition defined *inflammageing*. A couple of years ago, a seminal paper demonstrated that the pro-inflammatory cytokine TNF-α drives metabolic reprogramming of megakaryocytes, platelet mitochondrial dysfunction, and platelet hyper-reactivity as part of normal aging in humans and mice [57]. In this study, the sex differences were not examined. However, it is known that the aging process affects platelet function differently among sexes [58] and studies in other cells have shown that sex hormones modify the bioavailability of TNF-α [59]; thus, there is likely a combinatorial effect between age and sex.

Sexual dimorphism in the gut microbiota composition is also well documented. The microbiome shapes the immune system and influences platelet responsiveness, thus could be driving the sex differences in vascular disease. Trimethylamine N-oxide (TMAO), a metabolite derived from the gut microbiota, is an independent predictor of CVD, enhances platelet activation and promotes systemic inflammation [60]. However, only few studies so far have reported the sex differences in the level of TMAO or other gut-derived metabolites that may affect platelet and vascular homeostasis. Improved knowledge on this topic could be exploited to design dietary interventions to improve the microbiome and vascular health in a sex-specific manner.

Mental stress is an independent risk factors for CVD and women are more susceptible to stress and depression. A link that could explain the comorbidity between these two conditions is serotonin that is elevated in subjects with either mental stress and/or CVD and binds platelets from depressed patients with greater avidity [61]. Estradiol causes an increase in the production, transmission, and availability of serotonin (reviewed by Steiner et al. [62]), thus could explain the sexual dimorphism in both conditions.

## Sex differences in vascular disease

Vascular disease is a sexually dimorphic disease, but the interplay between platelets and sex in the pathogenesis of each disease is different and far to be elucidated. Indeed, two landmark clinical trials identified an opposite effect of aspirin in reducing myocardial infarction (MI) and ischemic stroke in men and women. The *Physician’s Health Study* showed that aspirin lowers the risk of MI but not of stroke in men [63]. The *Women’s Health Study* demonstrated that in women aspirin lowers the risk of ischemic stroke without affecting the risk of MI or death from cardiovascular causes [64].

### Sex differences in atherosclerosis

Population studies demonstrate that men grow more plaques than women and experience more atherothrombotic events, at least up to the age of 60–80 years, when women catch up and surpass men. Carotid artery intima-media thickness, a surrogate marker of early atherogenesis, is higher in men, and becomes comparable among sexes only after 75 years of age [65]. The reason for the delayed atherogenesis and the lower plaque burden in women is not known (Table 1). Women display a higher platelet reactivity, have more platelet-derived P-selectin-positive extracellular vesicles in circulation [66], and have higher serum levels of platelet-derived chemokines such as RANTES [67]. However, the pro-atherogenic effect of platelets is probably counterbalanced by the fact that pre-menopausal women suffer less than men of dyslipidaemia and hypertension [68], and have higher concentrations of estradiol that has vasoprotective and anti-inflammatory effects (reviewed by Xing et al. [69]).

**Table 1:** Sex differences in atherosclerosis in relation to platelet reactivity and other cardiovascular risk factors

♀		♂
+	Platelet reactivity	−
+	Extracellular vesicles	−
+	Platelet-derived chemokines	−
−	Dyslipidaemia	+
−	Hypertension	+
−	Carotid Intima thickness	+
−	Plaque number	+
−	Plaque area	+
+	Plaque stenosis	−
−	Plaque instability	+
−	Plaque rupture	+
+	Plaque erosion	−
−	Plaque Macrophage infiltration	+
=	Plaque T cell infiltration	=
nd	NETs	nd

Plaque morphology and composition are also different between males and females [65]. Men have a larger plaque area that correlates with a higher rate of cardiac events, while women display greater stenosis. Men and women appear to have comparable number of T cells in the plaques [70], but men have more macrophage infiltrates compared to women [71], and express more metalloproteinases in the plaque, conditions that make them more unstable and susceptible to plaque rupture. Conversely, women display more stable plaques that are more prone to surface erosion, rather than rupture. Different inflammatory pathways underlie plaque rupture and plaque erosion. NETs have been detected in the eroded plaques and participate in endothelial denudation [72]. Platelets from women have a higher propensity to bind neutrophils [73] than those of men. Thus, the increased platelet reactivity in women could explain, at least in part, the increased incidence of plaque erosion in women. The EROSION study has indeed shown that anti-platelet drugs without stenting is a safe treatment for patients with plaque erosion [74]. Future studies need to investigate the interplay of platelets with the different inflammatory pathways underlying plaque rupture and plaque erosion to tailor more precise treatments.

### Sex differences in coronary artery disease

Ischemic heart disease (IHD) is not always the consequence of obstructive CAD. Non-obstructive CAD, defined as < 50% stenosis of any epicardial coronary artery, is more common in young women with coronary vasomotor disorders or coronary microvascular dysfunction (CMD) (reviewed by Waheed et al. [75]). A recent study from our group has shown that in patients with IHD, an increased testosterone/estradiol ratio associates with poor outcome and with elevated concentrations of thromboxane A_2_ [76]. Thromboxane A_2_ induces vasoconstriction and could facilitate *ischemia with non-obstructed coronary artery disease* (INOCA) and *myocardial infarction with non-obstructed coronary artery disease* (MINOCA). Platelets are also the main source of serotonin that can induce microvascular constriction with minimal effects on epicardial coronary arteries [77] and was recently identified as a sensitive marker of CMD in patients with angina and non-obstructive CAD [78]. Serotonin levels in platelets are regulated by sex and age and are greater in young women [79], that are also more susceptible to non-obstructive CAD. Moreover, serotonin is associated with depression and mental stress, that in turn is associated with CMD and coronary vasospasm, especially in women [80]. However, alterations of the vascular tone are not the only mechanisms by which platelets could affect non-obstructive IHD in a sex-specific manner. Subjects with CMD have an elevated inflammatory state and serotonin has many immunomodulatory functions. Understanding the specific inflammatory signature and the platelet-immune interactions that characterize non-obstructive CAD may help us further dissect the underlying mechanism and understand why it is more prevalent in younger women.

### Sex differences in stroke

Stroke is the third leading cause of death for women, but the fifth for men [81]. Women have an overall incidence of ischemic stroke lower than men but display a higher incidence below 35 years of age [82] and above 85 years of age [83] and a higher mortality rate. Interestingly, risk factors are different among sexes and males and females suffer from different types of strokes [84]. Women are more likely to have cardioembolic strokes due to atrial fibrillation; men suffer more of atherothrombotic and lacunar strokes (mostly of microvascular origin). Moreover, women experience atypical stroke symptoms compared to men and for this reason fail to seek medical attention promptly and are more likely to suffer a greater brain damage. Women stroke survivors have worse functional recovery and greater long-term disability and handicap compared to men [85].

The sex differences in the type of stroke, clinical presentation and long-term consequences suggest that different pathogenic mechanisms cause the initial ischemia and stroke progression in males and females. Data from large clinical trials indicate that aspirin is effective in lowering the risk of ischemic stroke in women [64], but not in men [63], suggesting that platelets may play a distinct role among the two. Unfortunately, the preclinical studies dissecting the molecular mechanisms of stroke have used mostly male mice. A sub-group analysis in a recent study on ischemia/reperfusion injury after recanalization in mice, shows that both males and females benefit from the genetic deletion of CD84, suggesting that the crosstalk between platelets and T cells contributes to stroke progression in both sexes. However, only male CD84-knockout mice have a significantly improved neurological outcome [25] and female mice seem to have greater infarct size on average. More studies are needed to validate these observations and understand the sex-specific molecular mechanisms of ischemic stroke.

## Translational implications

The reason behind the sex differences in vascular disease is multifactorial. In this review, we have outlined the contribution of platelets to the pathogenesis of vascular disease, and we have summarized some of the mechanisms by which the platelet phenotype and function contribute to sexual dimorphism in vascular disease.

Understanding the sex differences at the platelet–vascular interface is clinically relevant as it will enable: (1) to design new strategies that prevent the detrimental effects of the immune-modulatory function of platelets taking sex into account, and (2) to evaluate if sex-specific antiplatelet drug regimens should be used to reduce the risk not only of thrombosis but also of vascular disease progression.
